# Supplementary material for: Cooperative unfolding of distinctive mechanoreceptor domains transduces force into signals
Source: eLife. 2016 Jul 19;5:e15447. doi: 10.7554/eLife.15447 (PMC5021522; doi:10.7554/eLife.15447)
Supplement: Figure 3—source data 1. — (A) Decision rules for and statistical summary of GPIbα domain unfolding in force-clamp experiment mode. Criteria for deciding whether or not (+ or −) and which (LRRD, MSD, or both) GPIbα domain(s) was (were) unfolded are based on BFP profile signatures and the unfolding lengths. YES = observed, NO = not observed. NA = not applicable. (B) Related to Figure 3F–H. Evaluation of LRRD and MSD unfolding cooperativity. All probabilities were calculated from occurrence data in (A). Observed joint probabilities were compared to their predicted counterparts based on the assumption that LRRD and MSD unfolded independently. For example, in 'WT A1 vs. Platelet' under 25 pN: The probability of LRRD unfolding is P(LRRD) = 3.4% + 6.9% + 2.76% = 13.06%. The probability of MSD unfolding is P(MSD) = 7.6% + 13.8% + 6.9% + 2.76% = 31.06%. The probability of MSD ramped unfolding is P(MSD, ramp) = 7.6% + 2.76% = 10.36%. The probability of MSD clamped unfolding is P(MSD, clamp) = 13.8% + 6.9% = 20.7%. DOI: http://dx.doi.org/10.7554/eLife.15447.011 [file elife-15447-fig3-data1.docx]

|  | **Clamped Force (pN)** | **Unfolding signatures** | | **Decision rules** | | **Number of observations and occurrence frequency** | |
| --- | --- | --- | --- | --- | --- | --- | --- |
|  |  | Ramping phase | Clamping phase | LRRD | MSD |  |  |
| **WT A1 vs. Platelet** | 10 | NO | NO | - | - | 115 | 93.50% |
|  |  | <25nm | NO | - | + | 1 | 0.81% |
|  |  | NO | <25nm | - | + | 7 | 5.69% |
|  | 25 | NO | NO | - | - | 95 | 65.52% |
|  |  | 25-56 nm | NO | + | - | 11 | 7.59% |
|  |  | <25nm | NO | - | + | 5 | 3.45% |
|  |  | NO | <25nm | - | + | 20 | 13.79% |
|  |  | <56nm | <25nm | + | + | 10 | 6.90% |
|  |  | >56nm | NO | + | + | 4 | 2.76% |
|  | 40 | NO | NO | - | - | 60 | 61.86% |
|  |  | 25-56 nm | NO | + | - | 10 | 10.31% |
|  |  | <25nm | NO | - | + | 8 | 8.25% |
|  |  | NO | <25nm | - | + | 10 | 10.31% |
|  |  | <56nm | <25nm | + | + | 6 | 6.19% |
|  |  | >56nm | NO | + | + | 3 | 3.09% |
|  | 60 | NO | NO | - | - | 45 | 60.81% |
|  |  | 25-56 nm | NO | + | - | 10 | 13.51% |
|  |  | <25nm | NO | - | + | 7 | 9.46% |
|  |  | NO | <25nm | - | + | 6 | 8.11% |
|  |  | <56nm | <25nm | + | + | 3 | 4.05% |
|  |  | >56nm | NO | + | + | 3 | 4.05% |
| **R1450E vs. Platelet** | 10 | NO | NO | - | - | 80 | 85.11% |
|  |  | 25-56 nm | NO | + | - | 0 | 0.00% |
|  |  | <25nm | NO | - | + | 1 | 1.06% |
|  |  | NO | <25nm | - | + | 13 | 13.83% |
|  |  | <56nm | <25nm | + | + | 0 | 0.00% |
|  |  | >56nm | NO | + | + | 0 | 0.00% |
|  | 25 | NO | NO | - | - | 93 | 74.40% |
|  |  | 25-56 nm | NO | + | - | 10 | 8.00% |
|  |  | <25nm | NO | - | + | 8 | 6.40% |
|  |  | NO | <25nm | - | + | 12 | 9.60% |
|  |  | <56nm | <25nm | + | + | 0 | 0.00% |
|  |  | >56nm | NO | + | + | 2 | 1.60% |
|  | 40 | NO | NO | - | - | 81 | 69.83% |
|  |  | 25-56 nm | NO | + | - | 13 | 11.21% |
|  |  | <25nm | NO | - | + | 12 | 10.34% |
|  |  | NO | <25nm | - | + | 7 | 6.03% |
|  |  | <56nm | <25nm | + | + | 0 | 0.00% |
|  |  | >56nm | NO | + | + | 3 | 2.59% |
|  | 60 | NO | NO | - | - | 45 | 68.18% |
|  |  | 25-56 nm | NO | + | - | 8 | 12.12% |
|  |  | <25nm | NO | - | + | 8 | 12.12% |
|  |  | NO | <25nm | - | + | 3 | 4.55% |
|  |  | <56nm | <25nm | + | + | 0 | 0.00% |
|  |  | >56nm | NO | + | + | 2 | 3.03% |

**Figure 3-Source Data 1A. Decision rules for and statistical summary of GPIbα domain unfolding in force-clamp experiment mode.** Criteria for deciding whether or not (+ or -) and which (LRRD, MSD, or both) GPIbα domain(s) was (were) unfolded are based on BFP profile signatures and the unfolding length. YES = observed, NO = not observed. NA = not applicable.

|  | **Clamped Force (pN)** | **Unfolding** | **Observed probability** | **Compared to** | **Predicted probability** | **Fold increase** |
| --- | --- | --- | --- | --- | --- | --- |
| **WT A1 vs. Platelet** | **10** | LRRD +,  MSD + | 0.00% | P_(MSD)_ * P_(LRRD)_ | 0.00% | 0 |
|  |  | LRRD +,  MSD ramped + | 0.00% | P_(MSD, ramp)_ * P_(LRRD)_ | 0.00% | 0 |
|  |  | LRRD +,  MSD clamped + | 0.00% | P_(MSD, clamp)_ * P_(LRRD)_ | 0.00% | 0 |
|  | **25** | LRRD +,  MSD + | 9.66% | P_(MSD)_ * P_(LRRD)_ | 4.65% | 1.1 |
|  |  | LRRD +,  MSD ramped + | 2.76% | P_(MSD, ramp)_ * P_(LRRD)_ | 1.07% | 1.6 |
|  |  | LRRD +,  MSD clamped + | 6.90% | P_(MSD, clamp)_ * P_(LRRD)_ | 3.58% | 0.9 |
|  | **40** | LRRD +,  MSD + | 9.28% | P_(MSD)_ * P_(LRRD)_ | 5.45% | 0.70 |
|  |  | LRRD +,  MSD ramped + | 4.12% | P_(MSD, ramp)_ * P_(LRRD)_ | 2.42% | 0.70 |
|  |  | LRRD +,  MSD clamped + | 5.16% | P_(MSD, clamp)_ * P_(LRRD)_ | 3.03% | 0.70 |
|  | **60** | LRRD +,  MSD + | 8.11% | P_(MSD)_ * P_(LRRD)_ | 5.56% | 0.46 |
|  |  | LRRD +,  MSD ramped + | 5.41% | P_(MSD, ramp)_ * P_(LRRD)_ | 3.21% | 0.68 |
|  |  | LRRD +,  MSD clamped + | 2.70% | P_(MSD, clamp)_ * P_(LRRD)_ | 2.34% | 0.15 |
| **R1450E vs. Platelet** | **10** | LRRD +,  MSD + | 0.00% | P_(MSD)_ * P_(LRRD)_ | 0.00% | 0 |
|  |  | LRRD +,  MSD ramped + | 0.00% | P_(MSD, ramp)_ * P_(LRRD)_ | 0.00% | 0 |
|  |  | LRRD +,  MSD clamped + | 0.00% | P_(MSD, clamp)_ * P_(LRRD)_ | 0.00% | 0 |
|  | **25** | LRRD +,  MSD + | 1.60% | P_(MSD)_ * P_(LRRD)_ | 1.69% | -0.05 |
|  |  | LRRD +,  MSD ramped + | 1.60% | P_(MSD, ramp)_ * P_(LRRD)_ | 0.77% | 1.08 |
|  |  | LRRD +,  MSD clamped + | 0% | P_(MSD, clamp)_ * P_(LRRD)_ | 0.92% | -1.00 |
|  | **40** | LRRD +,  MSD + | 2.59% | P_(MSD)_ * P_(LRRD)_ | 2.62% | -0.01 |
|  |  | LRRD +,  MSD ramped + | 2.59% | P_(MSD, ramp)_ * P_(LRRD)_ | 1.78% | 0.45 |
|  |  | LRRD +,  MSD clamped + | 0.00% | P_(MSD, clamp)_ * P_(LRRD)_ | 0.83% | -1.00 |
|  | **60** | LRRD +,  MSD + | 3.03% | P_(MSD)_ * P_(LRRD)_ | 2.98% | 0.02 |
|  |  | LRRD +,  MSD ramped + | 3.03% | P_(MSD, ramp)_ * P_(LRRD)_ | 2.30% | 0.32 |
|  |  | LRRD +,  MSD clamped + | 0.00% | P_(MSD, clamp)_ * P_(LRRD)_ | 0.69% | -1.00 |

**Figure 3-Source Data 1B. Related to Figure 3F-H. Evaluation of LRRD and MSD unfolding cooperativity.** All probabilities were calculated from occurrence data in Supplementary Table 1. Observed joint probabilities were compared to their predicted counterparts based on the assumption that LRRD and MSD unfolded independently. For example, in “WT A1 vs. Platelet” under 25 pN: The probability of LRRD unfolding is P_(LRRD)_ = 3.4% + 6.9% + 2.76% =13.06%. The probability of MSD unfolding is P_(MSD)_ = 7.6% + 13.8% + 6.9% + 2.76% =31.06%. The probability of MSD ramped unfolding is P_(MSD, ramp)_ = 7.6% + 2.76% =10.36%. The probability of MSD clamped unfolding is P_(MSD, clamp)_ = 13.8% + 6.9% =20.7%.
