# Supplementary material for: Cooperative unfolding of distinctive mechanoreceptor domains transduces force into signals
Source: eLife. 2016 Jul 19;5:e15447. doi: 10.7554/eLife.15447 (PMC5021522; doi:10.7554/eLife.15447)
Supplement: Figure 4—source data 1. — w1 represents the fraction of binding events that dissociate with the off-rate k1. The fraction of events that dissociate with the off-rate of k2 is simply calculated as w2 = 1-w1. NA = not applicable. DOI: http://dx.doi.org/10.7554/eLife.15447.014 [file elife-15447-fig4-data1.docx]

|  | **MSD unfolding** | **A1**–**GPIbα bond kinetic parameters by model fitting.** | | | |
| --- | --- | --- | --- | --- | --- |
| **Force (pN)** | ***k*_u_ (s^-1^)** | **LRRD unfolding** | ***w*_1_** | ***k*_1_ (s^-1^)** | ***k*_2_ (s^-1^)** |
|  | **WT** | | | | |
| **10** | 0.705 | **-** | 0.712 | 71.33 | 2.711 |
| **25** | 0.870 | **-** | 0.703 | 23.80 | 0.486 |
|  |  | **+** | 0.263 | 8.430 | 0.597 |
| **40** | 2.952 | **-** | 0.665 | 70.56 | 3.826 |
|  |  | **+** | 0.448 | 25.67 | 1.764 |
| **60** | 9.992 | **-** | 0.910 | 75.42 | 6.236 |
|  |  | **+** | 0.775 | 88.10 | 3.178 |
|  | **R1450E** | | | | |
| **10** | 0.362 | **-** | 0.8354 | 10.14 | 0.144 |
| **25** | 1.693 | **-** | 0.8416 | 22.60 | 1.776 |
| **40** | 3.183 | **-** | 0.9336 | 44.88 | 5.827 |
| **60** | 8.887 | **-** | 1 | 160.85 | NA |

**Figure 4-Source Data 1. MSD unfolding rates (*k*_u_) and the fraction (*w*_1_) and off-rates (*k*_1_, *k*_2_) of GPIbα binding to A1WT or A1R1450E under different forces.** *w*_1_ represents the fraction of binding events that dissociate with the off-rate *k*_1_. The fraction of events that dissociate with the off-rate of *k*_2_ is simply calculated as *w*_2_ = 1- *w*_1_. NA = not applicable.
